# Essential oils for managing *Pratylenchus penetrans* on Easter lilies

**DOI:** 10.21307/jofnem-2020-010

**Published:** 2020-03-17

**Authors:** B. B. Westerdahl, D. Giraud, L. J. Riddle, C. A. Anderson

**Affiliations:** 1 Department of Entomology and Nematology, University of California, Davis, CA 95616; 2 University of California Cooperative Extension, Eureka, CA 95501; 3 Easter Lily Research Foundation, Brookings, OR 97415

**Keywords:** 1,3-dichloropropene, Cinnamite, Duogard, EF300, EF400, Easter lily, Essential oils, Ethoprop, Fosthiazate, Lesion nematode, *Lilium longiflorum*, Management, Metam sodium, Phorate, *Pratylenchus penetrans.*

## Abstract

Easter lily bulbs for greenhouse forcing are produced in Del Norte County, California and Curry County, Oregon, USA. *Pratylenchus penetrans* infestation seriously affects growth of field grown bulbs. During two consecutive years of field trials containing 22 treatments, commercially prepared formulations of essential oils (EOs) were compared to an untreated control and to a standard chemical fumigant treatment (FU) (1,3-dichloropropene and metam sodium) applied preplant followed by phorate (PH) at planting to determine their value in the management of lesion nematode, and in improving plant health. The EO products Duogard, EF400, EF300, and Cinnamite were tested as preplant dips to bulblet planting stock. The treated bulblets were tested either alone, in combination with PH at-planting, at planting following FU or in combination with PH at planting following FU. The organophosphates ethoprop and fosthiazate were also tested either alone, or at a reduced rate in combination with a reduced rate of PH. With respect to bulb circumference, ten treatments consistently outperformed the control. In consecutive years, three treatments had healthier looking roots than the control. At harvest, levels of lesion nematode within roots were consistently lower in nine treatments. EOs were beneficial in mitigating nematode damage.

Easter lilies have been the most important crop in Humboldt and Del Norte counties of California (CA) and Curry County, Oregon (OR), USA since the early 1940s. This is the only area of the United States where Easter lily (*Lilium longiflorum* Thunb.) bulbs are grown commercially. Approximately 250 ha of bulbs are grown each year in a three to six-year rotation with pastures for cattle grazing; thus approximately 2,400 ha are required for the cropping system. Every year more than 11mn bulbs are sold for forcing. The industry is one of the area’s largest employers. Yearly farm gate value of the crop is approximately $9.6mn. Bulbs are sold to greenhouse operations nationwide for forcing to produce flowering plants at Easter. Quality of field grown bulbs is based on bulb circumference and appearance, with only bulbs with white scales that have plentiful roots being saleable. Bulbs are grown for 2 to 4 years before they are large enough for sale. Land is prepared in May, fumigated in July, bulblets are planted from August through October, and bulbs are harvested the following August through October (Roberts et al., 1985). Bulbs not reaching marketable size in two consecutive growing field years are replanted for additional years. Basic principles of nematode control dictate that effective management requires a combination of clean planting stock and clean soil, as well as an understanding of the biology of the pests involved. Since 1976, Easter lily growers have actively sought alternatives to the use of pesticides for management of the crops’ major pest, the lesion nematode (*Pratylenchus penetrans*) (Westerdahl et al., 2003).

Essential oils (EOs) are complex mixtures of volatiles, mainly products of plant secondary metabolism. Common components include terpenes, mono- and sesquiterpenes, and phenolic compounds, such as phenylpropanoids. They are generally biodegradable, have low toxicity to mammals and do not accumulate in the environment (Figueiredo et al., 2008). Chitwood (2002) reviewed the research on the effect of EOs on plant parasitic nematodes. Effects reported in several papers utilizing a variety of EOs against root-knot nematode included mortality of juveniles; and inhibition of mobility, hatching, infection and gall formation.

Since the review by Chitwood (2002), additional research with EOs on plant parasitic nematodes has shown promising results. A number of papers have looked at EOs fractionated into their component parts. For example, Faria et al. (2013) working with fractions of EOs for management of the pinewood nematode, *Bursaphelenchus xylophilus*, concluded that the unfractionated oils were more effective than their individual components. In an examination of plant EOs from 43 plant species Il-Kwon et al. (2005) found those from garlic and cinnamon to be the most effective against *Bursaphelenchus xylophilus*.

Seasonal weather patterns greatly affect quality and size of bulbs even in the absence of nematode pests. For example, “Nellie White” bulbs produced one year can be more than double the size of those produced in another year (Roberts et al., 1985). Trials conducted at the Easter Lily Research Foundation (ELRF) Station rotate through four different fields. Therefore, in addition to weather variation, there is additional variability in soil characteristics and nematode population levels. Even the standard products utilized by growers have shown year-to-year variability working better in some years than others (L.J. Riddle, pers. comm.).

The primary objective of this study was to evaluate preplant treatments of Easter lily bulblets with commercially prepared formulations of four EO products to an untreated control (UC), and a chemical standard to determine their value in the management of lesion nematode, and the subsequent growth of bulbs. A secondary objective was to evaluate the effectiveness of the organophospate nematicides ethoprop and fosthiazate used either alone, or in combination with a reduced rate of phorate.

## Materials and methods

During two years of field trials conducted at the ELRF Research Station in Brookings, OR, commercially prepared formulations of four EO products were compared to an UC and a standard chemical treatment for management of *Pratylenchus penetrans*. Nematodes were identified by both morphometric and molecular methods (Qiu et al., 2005). Each trial consisted of 22 treatments. The description of the nematicide treatments, and abbreviations used in the data tables are provided in Table 1. A standard chemical treatment of 1,3-dichloropropene (Telone II, Dow AgroSciences, Indianapolis, IN) plus metam sodium (Vapam, Amvac, Los Angeles, CA) (FU) was applied preplant followed by phorate (Thimet, Amvac, Los Angeles, CA) (PH9) at planting (FUPH9). The EO products Duogard (DU), EF400 (EF4), EF300 (EF3), all from USAgriTech (Paso Robles, CA), and Cinnamite (CI) (Mycotech, Butte, MT) were tested as preplant dips to bulblet planting stock. EF300 is an insecticide product containing the EOs of rosemary (5.4%), sesame (5.2%), peppermint (4.6%), thyme (3.7%) and cinnamon (3.5%) plus malic acid (3.3%). EF400 is a fungicide composed of the EOs of clove (6.2%), rosemary (6.1%) and peppermint (5.7%) plus malic acid (3.3%). Duogard is recommended for both insect and disease control and contains the EOs rosemary (6%), peppermint (5.5%), clove (4.3%), sesame (1.6%), cinnamon (1.2%) and thyme (1.2%) plus malic acid (3.3%). These EO products were tested either alone, in combination with PH9 at-planting, at planting following FU, or in combination with PH9 at planting following FU. In the first trial, bulblet dip treatments were 1/2% of product by volume. In the second trial, the CI dip was 1/2% of product by volume and the other products were at 2% of product by volume. The organophosphate ethoprop (Mocap, Amvac, Los Angeles, CA) was tested either alone (ET13); in combination with FU; at a reduced rate (ET6) combined with a reduced rate of phorate (PH5); or at a reduced rate in combination with FU and PH5. The organophosphate fosthiazate (Nemathorin, Syngenta International AG, Basel, Switzerland) was applied with FU or alone at planting followed by second application in the spring (FUFO2X, FO2X); at planting in combination with a reduced rate of PH; or in combination with FU and a reduced rate of PH at planting. Logistics did not permit testing all products in all combinations.

**Table 1.** Description of treatments and abbreviations used in text and tables.

		Bulblet dip (d),	ap, pp
	Preplant	At planting (ap), or	Rate
Abbreviation^a^	Treatment (FU)	Post plant (pp)	kg a.i./ha^b^
FUPH9	Yes	Phorate (ap)	9
FUET13	Yes	Ethoprop (ap)	13.4
FU	Yes	none	None
FUDUPH9	Yes	DuoGard (d) Phorate (ap)	9
FUDU	Yes	DuoGard (d)	None
FUEF4PH9	Yes	EF400 (d) Phorate (ap)	9
FUEF4	Yes	EF400 (d)	None
FUEF3PH9	Yes	EF300 (d) Phorate (ap)	9
FUEF3	Yes	EF300 (d)	None
FUPH5FO	Yes	Phorate (ap) Fosthiazate (ap)	4.5–4.4
FUPH5ET8	Yes	Phorate (ap) Ethoprop (ap)	4.5–7.8
FUFO2X	Yes	Fosthiazate (ap) (pp)	4.4–4.4
FUCIPH9	Yes	Cinnamite (d) Phorate (ap)	9
FUCI	Yes	Cinnamite (d)	None
CIPH9	No	Cinnamite (d) Phorate (ap)	9
CI	No	Cinnamite (d)	None
PH5FO	No	Phorate (ap) Fosthiazate (ap)	4.5–4.4
PH5ET8	No	Thimet (ap) Ethoprop (ap)	4.5–7.8
FO2X	No	Fosthiazate (ap) (pp)	4.4–4.4
PH9	No	Phorate (ap)	9
ET13	No	Ethoprop (ap)	13.4
UC	No	None	None

Notes: ^a^FU = Preplant treatment of 1,3-Dichloropropene at 428 kg a.i./ha plus metam sodium at 407 kg a.i./ha; ^b^kg a.i./ha is expressed as the amount of product that was actually applied.

Planting stock was “Nellie White” bulblets hand graded from scale bed production of the previous year’s crop and weighed approximately 7 g each. For all treatments, bulblets were dipped for 1 hr at 12°C in a freshly made fungicide solution of 0.72 kg a.i. pentachloronitrobenzene (Terraclor 400, 40% PCNB, Uniroyal Chemical Company, Middlebury, CT), 0.95 kg a.i. tetramethylthiuram disulfide (42-S Thiram, Gustafson, Plano, TX), 0.81 kg a.i. carboxin (Vitavax-34, Gustafson, Plano, TX), 0.052 kg a.i. thiophanate-methyl (SysTec, Regal Chemical Company, Alpharetta, GA), and 1% M-Pede (Dow AgroSciences Indianopolis, IN), per 379 liters of water and planted within 24 hr of treatment. For EO treatments, the products were added to the fungicide dip solution. Following treatments, levels of nematodes in bulblets were reduced compared to UC but not eliminated.

Bulblets were planted in October each year in a field that is managed to maintain a population of *P. penetrans* by rotating lilies with clover. Bulblets were hand planted and harvested. Tractor drawn implements were used for land preparation, bed formation and digging of bulbs (which were then picked up by hand). All cultural operations were done with great precision to ensure the integrity of the individual plots. Treatment effects were evaluated through assessment of crop quality, and nematode population densities in soil and root samples. Soil samples from each replicate were taken at harvest in September to a depth of 30-cm with a 2.5-cm-diameter soil tube (8 cores per sample).

Two trials were conducted in consecutive years in a randomized complete block design with three replicates of 75 bulblets each per treatment. Plots were one row (1.02-m) wide by 6-m long. The circumference of each harvested bulb was measured and means per plot presented. Additional variables analyzed were percent survival, weight of foliage from five randomly selected plants, a visual rating of roots from 1 (poor) to 10 (excellent) as a measure of root health, and weight of bulblets from the same five plants. Following harvest, soil and root samples were transported to the University of California Davis Cooperative Extension Nematode Diagnostic Laboratory. Within one week, nematodes were extracted from soil using a modified semiautomatic elutriator and sugar flotation technique (Byrd et al., 1976). Nematodes were extracted from roots cut from the base of five bulbs per replicate. Roots were washed, weighed and placed in an intermittent misting chamber for 72 hr. Extracted nematodes were identified, then counted using a stereoscopic microscope. Because the soil nematode population in the UC in Trial 1 was double that in Trial 2, results were evaluated for each trial separately using analysis of variance followed by LSD testing at *p* ≤ 0.05, and by linear regression (SuperAnova, Berkeley, CA) rather than combining the trials for analysis.

## Results

In the first trial, 13 treatments, including all EO treatments, but not the standard FUPH9 had a greater bulb circumference than UC (*p* ≤   0.05) (Table 2). In the second trial, 16 treatments including the standard, and seven EO treatments had a greater bulb circumference than UC (*p* ≤ 0.05). Ten treatments, including seven EO treatments, outperformed the control in both years (*p* ≤ 0.05). In the first trial, 10 treatments, including the four EO CI treatments, had healthier looking roots than UC (*p* ≤ 0.05). In the second trial, six treatments, including three EO treatments had healthier looking roots than UC (*p* ≤ 0.05). In both trials, three treatments, including one EO treatment, had healthier looking roots than UC (*p* ≤ 0.05).

**Table 2.** Effect of treatments on bulb circumference and root health.

	Bulb circumference (cm)		Root rating^a^	
Treatment	Trial 1	Trial 2	Trial 1	Trial 2
FUPH9	14.0	13.6*	5.7	4.3
FUET13	14.1	14.3*	6.3*	3.7
FU	14.1	14.2*	4.3	5.7
FUDUPH9	14.6*	14.3*	4.7	4.3
FUDU	14.7*	14.4*	4.3	5.3
FUEF4PH9	14.5*	14.1*	5.2	5.3
FUEF4	14.9*	14.6*	3.3	6.3*
FUEF3PH9	14.6*	14.2*	4.0	6.3*
FUEF3	14.7*	14.2*	4.3	5.3
FUPH5FO	14.6*	13.6*	7.0*	7.3*
FUPH5ET8	14.6*	13.7*	5.3	7.0*
FUFO2X	14.2	14.0*	6.3*	6.8*
FUCIPH9	14.5*	14.1*	7.3*	6.0*
FUCI	14.7*	14.0*	6.7*	5.7
CIPH9	14.6*	13.0	7.0*	4.8
CI	14.5*	12.2	7.0*	3.0
PH5FO	14.4*	13.5	7.7*	4.7
PH5ET8	13.9	13.1	6.3*	4.3
FO2X	13.7	13.6*	6.3*	4.7
PH9	13.1	13.3	4.7	5.7
ET13	13.4	13.6*	5.3	4.3
UC	13.4	12.8	4.0	3.7
LSD	0.927	0.744	1.990	2.085

Notes: Data are means of three replicates. *Significantly different from UC at p ≤ 0.05; ^a^root rating was subjective on a scale from 1 = poor to 10 = healthy looking.

In the first trial, no treatments had greater survival than UC, and one treatment had lower survival (*p* ≤ 0.05) (Table 3). In the second trial, four treatments including the standard, and three EO treatments, had greater bulb survival than UC (*p* ≤ 0.05). Six treatments in the first trial, including three EO treatments, had a greater foliage weight at harvest than UC (*p* ≤ 0.05). In total, 18 treatments in the second trial, including the standard treatment, and 8 EO treatments, had a greater foliage weight at harvest than UC (*p* ≤ 0.05). In both trials, four treatments, including two EO treatments had a greater foliage weight than UC (*p* ≤ 0.05). In the first trial, the standard and five EO treatments produced a greater weight of stem bulblets than UC (*p* ≤ 0.05). Ten treatments in the second trial, including seven EO treatments, produced a greater weight of stem bulblets than UC (*p* ≤ 0.05). Three EO treatments were the only treatments consistently greater than UC over the two years of trials (*p* ≤ 0.05).

**Table 3.** Effects of treatments on survival and growth of Easter lilies.

			Foliage Weight		Bulblet Weight	
	Bulb survival (%)^a^		(5 plants) (g)		(5 plants) (g)	
Treatment	Trial 1	Trial 2	Trial 1	Trial 2	Trial 1	Trial 2
FUPH9	98	96*	111.3	129.3*	2.9*	2.7
FUET13	96	87	137.3*	117.3*	2.7	2.4
FU	94	93	93.3	102.7*	2.1	3.1*
FUDUPH9	100	95	103.3	96.7*	2.4	2.8*
FUDU	93	97*	142.0*	122.0*	3.3*	2.9*
FUEF4PH9	96	91	124.0	91.3*	3.2*	3.0*
FUEF4	95	100*	100.7	110.7*	2.3	2.7
FUEF3PH9	95	89	122.0	132.0*	3.6*	2.7
FUEF3	98	95	118.7	104.0*	2.6	4.4*
FUPH5FO	94	91	126.3	111.3*	2.2	3.3*
FUPH5ET8	96	94	128.0	104.0*	2.3	3.3*
FUFO2X	96	96	120.3	119.3*	2.2	2.7
FUCIPH9	72*	94	133.3*	107.3*	2.5	3.2*
FUCI	96	96*	118.7	96.0*	3.2*	2.9*
CIPH9	95	85	147.7*	73.3	3.3*	1.5
CI	94	87	122.7	56.7	2.2	1.5
PH5FO	96	90	142.0*	95.3*	2.0	2.6
PH5ET8	91	86	132.0*	65.3	1.9	1.9
FO2X	92	88	109.7	103.3*	1.7	3.3*
PH9	90	92	91.3	88.7*	2.0	2.4
ET13	92	90	106.0	97.3*	1.6	2.6
UC	95	87	95.0	52.7	1.5	1.3
LSD	13.785	9.287	36.157	33.182	1.251	1.436

Notes: Data are means of 3 replicates. ^a^Bulb survival out of 75 planted per replicate; ^*^significantly different from UC at p ≤ 0.05.

Three treatments in the first trial, including the standard, but none in the second trial had a lower level of lesion nematode in soil at harvest than UC (*p* ≤ 0.05) (Table 4). Nine treatments in the first trial, including the standard and three EO treatments, had lower levels of lesion nematode within roots at harvest than UC (*p* ≤ 0.05). In total, 16 treatments in the second trial, including the standard, and eight EO treatments, had lower levels of lesion nematode within roots at harvest than UC (*p* ≤ 0.05). Nine treatments, including the standard and three EO treatments, performed consistently over the two years of trials (*p* ≤ 0.05).

**Table 4.** Effect of treatments on densities of *Pratylenchus penetrans* in soil and roots.

	*Pratylenchus penetrans*			
	Soil (n/1,000 cm)^3^		Roots (n/g)	
Treatment	Trial 1	Trial 2	Trial 1	Trial 2
FUPH9	16.7*	16.7	51.6*	16.5*
FUET13	116.7	33.3	42.2*	7.3*
FU	283.3	133.3	131.2	70.5*
FUDUPH9	300.0	16.7	72.9	10.6*
FUDU	283.3	66.7	63.9*	53.0*
FUEF4PH9	116.7	33.3	29.3*	15.5*
FUEF4	183.3	66.7	194.5	63.8*
FUEF3PH9	183.3	0.0	140.7	46.4*
FUEF3	333.3	50.0	250.9	33.9*
FUPH5FO	66.7	33.3	29.7*	0.5*
FUPH5ET8	150.0	50.0	56.9*	31.5*
FUFO2X	33.3*	0.0	18.3*	0.4*
FUCIPH9	33.3	16.7	21.2*	45.2*
FUCI	250.0	100.0	224.3	44.3*
CIPH9	350.0	33.3	230.8	175.2
CI	333.3	66.7	150.6	121.4
PH5FO	16.7*	33.3	16.4*	53.7*
PH5ET8	200.0	50.0	72.7	103.3
FO2X	83.3	16.7	75.0	29.7*
PH9	250.0	166.7	117.7	120.7
ET13	200.0	50.0	355.0	116.8
UC	266.7	133.3	256.2	157.4
LSD	319.6	135.136	185.75	72.328

Notes: Data are means of three replicates. ^*^Significantly different from UC at p ≤ 0.05.

Based on the overall frequency of statistically significant results (*p* ≤ 0.05) (Tables 2–4), with respect to nematode control, the FU treatment alone was less effective than the standard combination treatment FUPH9 but was similar with respect to the various plant growth factors evaluated. Using the same criteria, when used alone, PH9, ET13 and FO2X were less effective than when used in combination with FU, with respect to both nematode control and plant growth. FUFO2X and treatments with a reduced rate of PH combined with another organophosphate (FUPH5FO, FUPH5ET8, PH5FO, PH5ET8) provided slightly less effective nematode control, but equivalent or better overall growth than FUPH9, particularly with respect to bulb circumference and root rating.

In both trial 1 (*y* = 97.429757 + 0.7326846 × *x*, *p* ≤ 0.0015), and in trial 2 (*y* = 25.345391 + 0.462281 ×* x*, *p* ≤ 0.0109), there was a strong correlation between nematodes present in roots and in soil. However, in two years of trials, only 3 treatments out of 42 showed a significant nematode reduction in soil, as opposed to 25 treatments for roots. This indicates that it is important to analyze nematodes present in roots as opposed to only those in soil surrounding roots in order to judge the effectiveness of nematode control on Easter lilies.

Of the criteria evaluated, bulb circumference and root rating are the most important to growers. Trial 1 did not reveal any significant correlation between nematodes present in roots or soil and the growth characteristics evaluated. In Trial 2, there was good correlation between nematodes present in roots and bulb circumference (*y =* 14.2105 − 0.0077649 ×* x, p* ≤ 0.0007), root rating (*y =* 5.7628954 − 0.0093228 ×* x*, *p* ≤ 0.0531), bulb survival (*y =* 94.19439 − 0.0404346×*x*, *p* ≤ 0.0177), foliage weight (*y =* 117.51956 − 0.3103599 ×* x*, *p* ≤ 0.0001), and bulblet weight (*y =* 3.2723891 − 0.009709 × *x*, *p* ≤ 0.0002). A strong correlation was also revealed between nematodes present in soil and foliage weight (*y* = 110.35572−0.215416 ×* x*, *p* ≤ 0.0372).

## Discussion

Bulb circumference is the most important criteria for marketing bulbs. Based on this, all four of the EO treatments (DuoGard, EF400, EF300 and Cinnamite) significantly improved bulb circumference when used in combination with the fumigant. Addition of an organophosphate did not provide an increase in bulb circumference. Using either a fumigant or organophosate treatment alone did not consistently increase bulb circumference. The two most effective chemical combination treatments were fumigant plus phorate with fosthiazate, and fumigant plus phorate with ethoprop.

In the Easter lily cropping system, severe pest pressure resulting from both nematode infested soil and infected planting stock results in growers typically using a dual nematicide application consisting of a dual preplant fumigant treatment followed by an organophosphate at planting. Even the standard products utilized by growers have shown year-to-year variability working better in some years than others (L.J. Riddle, pers. comm.), as they did in the present trials. In previous trials testing CI (but not the other EO products), CI had shown good but inconsistent results when used alone (unpubl. data) and was again tested alone in these trials. Because of the previous inconsistent results with CI, we chose to test all the EO products in combination with a fumigant, or fumigant plus organophosphate to see if they could provide an improvement over or replace a component of the standard treatment.

Recent research has also shown that EOs have activity against insects and plant disease organisms and may also promote plant growth in the absence of pests (Gupta et al., 2011; Pingsheng et al., 2007). In a field trial, Abo-Elyousr et al. (2009) found that a mixture of EOs was less effective than oxamyl at controlling root-knot nematode on tomatoes but provided a significantly greater increase in yield than did oxamyl. It is likely that similar plant growth interactions are occurring in the present trials. However, the reductions in nematode populations that were obtained in our trials indicate that nematode control is an important component of the improvement in plant growth that was achieved as a result of the EO treatments.

In spite of the inherent variability in physical and biological factors, our trials demonstrated that the EO products improved bulb circumference, foliage weight, root health and reduced nematode pressure over the UC in multiple treatments in both years of the trials. In spite of the variability that naturally occurs in field trials conducted over multiple years, our results were fairly consistent. When used with FU but not in combination with PH9, EO products performed similarly to FUPH9 in both trials. Thus, these products show potential for use in conjunction with a preplant fumigant. This is an important finding for producers to meet their overall goal of reducing pesticide use.
